# Universal Behavior of Fractal Water Structures Observed in Various Gelation Mechanisms of Polymer Gels, Supramolecular Gels, and Cement Gels

**DOI:** 10.3390/gels9070506

**Published:** 2023-06-21

**Authors:** Shin Yagihara, Seiei Watanabe, Yuta Abe, Megumi Asano, Kenta Shimizu, Hironobu Saito, Yuko Maruyama, Rio Kita, Naoki Shinyashiki, Shyamal Kumar Kundu

**Affiliations:** 1 Department of Physics, School of Science, Tokai University, Hiratsuka-shi 259-1292, Japan; abe.yuta@jaea.go.jp (Y.A.); asanom@tcu.ac.jp (M.A.); hilonobu.saito@gmail.com (H.S.); greyukoen@gmail.com (Y.M.); rkita@keyaki.cc.u-tokai.ac.jp (R.K.); naoki-ko@keyaki.cc.u-tokai.ac.jp (N.S.); 2 Course of Physics, Graduate School of Science, Tokai University, Hiratsuka-shi 259-1292, Japan; seiei.watanabe@gmail.com (S.W.); dynamite724@gmail.com (K.S.); 3 Micro/Nano Technology Center, Tokai University, Hiratsuka-shi 259-1292, Japan; 4 Department of Physics, School of Basic and Applied Sciences, Galgotias University, Greater Noida 201306, India; kundu05@gmail.com

**Keywords:** gelation mechanism, dielectric relaxation, water structures, fractal analysis

## Abstract

So far, it has been difficult to directly compare diverse characteristic gelation mechanisms over different length and time scales. This paper presents a universal water structure analysis of several gels with different structures and gelation mechanisms including polymer gels, supramolecular gels composed of surfactant micelles, and cement gels. The spatial distribution of water molecules was analyzed at molecular level from a diagram of the relaxation times and their distribution parameters (*τ*–*β* diagrams) with our database of the 10 GHz process for a variety of aqueous systems. Polymer gels with volume phase transition showed a small decrease in the fractal dimension of the hydrogen bond network (HBN) with gelation. In supramolecular gels with rod micelle precursor with amphipathic molecules, both the elongation of the micelles and their cross-linking caused a reduction in the fractal dimension. Such a reduction was also found in cement gels. These results suggest that the HBN inevitably breaks at each length scale with relative increase in steric hindrance due to cross-linking, resulting in the fragmentation of collective structures of water molecules. The universal analysis using *τ–β* diagrams presented here has broad applicability as a method to characterize diverse gel structures and evaluate gelation processes.

## 1. Introduction

The common features of gelation are the presence of cross-linking points and the inclusion of solvent molecules at cross-linking points [1,2,3]. Therefore, any gel material has a structure similar to that of ordinary polymer gels at various spatial scales. On the other hand, regarding the gelation process, each molecular mechanism constructs the gel structure on various lengths and time scales, so the scales of observation and analysis are inevitably diversified.

**Figure 1:**
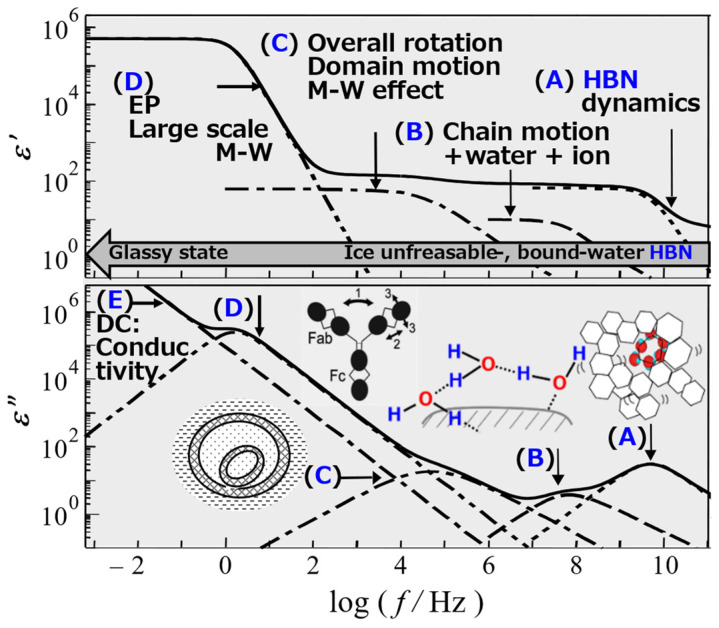
Relaxation processes reflect typical molecular mechanisms in aqueous systems. (**A**) 10 GHz process: dynamics of HBN: hydrogen bonding network (**B**) Dynamics of polymer chain + interacting water molecules + ion (**C**) Overall rotation, Domain motion, Maxwell–Wagner (M-W) effect (**D**) Large-scale M-W, EP: Electrode Polarization (**E**) DC conductivity.

Dielectric relaxation measurements allow us to observe the dynamic behavior of molecules from changes in polarization [4,5]. In particular, recent broadband dielectric spectroscopy (BDS) methods have demonstrated that the dynamic behavior of atoms, molecules, ions and their assemblies can be observed as multiple relaxation processes over a wide frequency range [6,7]. It is thus effective for analyzing fluctuations in hierarchical and heterogeneous structures characteristic of gel research and interactions between solvent and solute molecules. Therefore, observation and analysis by BDS can be one of the most effective physical measurement methods to analyze the dynamic structures and physical properties of gels. Figure 1 shows a schematic representation of these hierarchical structures and the molecular mechanisms of observable dielectric relaxation processes [7,8]. Practical applications require modification of the optimal number of relaxation processes and those mechanisms for each material and measurement condition involved.

In general, dielectric relaxation curves obtained from BDS measurements are analyzed as a superposition of such multiple relaxation processes as they reflect polarization phenomena appearing in each frequency region corresponding to the characteristic time. In order to identify the dynamic behavior of each relaxation phenomenon and analyze its dependence on physical conditions, three relaxation parameters, namely, the relaxation strength, the relaxation time, and the distribution parameter are often used to represent each relaxation process. These parameters are interpreted in terms of the physical meaning of effective dipole moments and their number densities, characteristic times of dipole correlations, and their distribution parameters, respectively [5,7,8]. Therefore, the BDS measurement technique is very useful for evaluating the mean and fluctuating dynamics at the molecular level that determine permeation and transport phenomena in gels and for understanding important physical implications [9,10,11,12,13]. Conventional BDS analysis defines the permittivity as a function of frequency and treats the relaxation time and its distribution parameters as mutually independent variables. On the other hand, Feldman et al. proposed an analytical method to describe the relationship between these variables by introducing the ergodic hypothesis and the spatial fractal dimension [14,15,16]. A more advanced analytical method has also been systematically reported [17,18,19]. In the present study, however, we focus on universality as an analysis of water structures with fewer restrictions and higher applicability; the former model is used. The mathematical expression for the relationship between the relaxation time and its distribution parameters relevant to this study is briefly introduced in Appendix A. In addition to the analytical method, an interpretation [20,21] that the 10 GHz process observed in hydrogen-bonded liquids reflects the dynamic behavior of hydrogen-bonding networks (HBNs) formed by water and interacting molecules, allowing us to discuss the spatial distribution information of water molecules [7,8,22].

Conventional dielectric spectroscopy treats the time correlation function between dipole moments or charges as relaxation processes and discusses dynamic behaviors and fluctuations of molecules and ions. Therefore, dielectric spectroscopy is essentially unable to directly observe the spatial structure and cannot analyze the spatial distribution of water molecules. Therefore, in the present work, the ergodic hypothesis and fractal spatio-temporal correlation were introduced with the meaning of the fractal spatial structure of the hydrogen bonding network formed by water molecules with other molecules and make it possible to analyze the spatial distribution of water. The method of analyzing the spatial distribution of water molecules at the molecular level is extremely rare and unique. Furthermore, this spatial distribution analysis of water molecules revealed that even with the same relaxation time distribution parameter value, the spatial distribution of water molecules differs as much as in solution systems and suspensions if the relaxation times are different [7,8]. This means that the reliability of the argument to infer structural heterogeneity from the relaxation time distribution parameters, which has been done in conventional analyses, is low.

The purpose of this study is to apply this unique and universal analysis to the gelation process of several types of gel structures and molecular mechanisms, and to clarify the common properties of gelation from the viewpoint of water structures. Gel samples used here are polymer gels [22,23,24,25,26,27], supramolecular gels [28,29,30], and cement gels [31,32,33,34,35], for which conventional relaxation parameter analysis has been already completed. However, as no data on cement samples with admixtures have been previously reported, conventional procedures for dielectric relaxation measurements and parameter analysis are also reported here.

In the case of aqueous gel systems, even if it is effective for elucidating diverse and complex higher-order structures due to interacting water molecules, dielectric properties are often hidden by high electrical conductivity and makes measurements and analyses difficult. However, high-frequency dielectric measurements for the 10 GHz process makes it possible to analyze water structures and dynamics for which conductivity affects less than the lower-frequency processes. In this study, we used the novel universal analysis described above to investigate how common structural features are expressed in several gel materials with widely different molecular mechanisms of gelation. Based on former results reported for polymer gels [22,23,24,25,26,27], supramolecular gels [28,29,30], and cement gels [31,32,33,34,35], we conducted a universal analysis of water structures in the molecular mechanism of these gelation processes. Unpublished data for cement gels used in part of this study are described in the respective sections of the present report.

## 2. HBN Fragmentation Model

**Figure 2:**
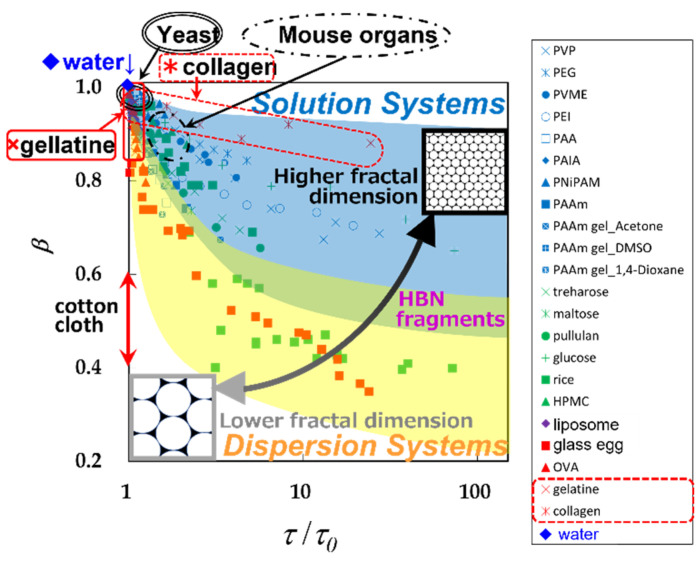
The *τ*–*β* diagrams of various hydrocolloids (extensive database) [14,15]. Commonly used abbreviations are used in the legend [38]. The yeast and mouse organ data regions are also added [8]. (Adapted from Yagihara et al. [38]).

Figure 2 shows plots of the relaxation time normalized by that for pure water, *τ*/*τ*_0_, for the 10 GHz process against the relaxation time distribution parameter, *β*, obtained so far for various materials. The distribution parameter used here is defined by the Cole–Cole equation [36] as:

(1) $$\varepsilon^{*}=\varepsilon^{\prime }-j\varepsilon^{\prime \prime }=\varepsilon_{\infty }+\frac{\Delta \varepsilon }{1+{\left(j\omega \tau \right)}^{\beta }}\;,\;\;\;\;\;\;\;\;\;\;\left(0<\beta \leq 1\right)\;,$$

where ε* is the complex dielectric constant with the real and imaginary parts, $\varepsilon^{\prime }$ and $\varepsilon^{\prime \prime }$, *j* is the imaginary unit, Δ*ε* ($=\varepsilon_{0}-\varepsilon_{\infty }$) is the relaxation strength, *ε*_0_ and *ε*_∞_ are the low and high-frequency limit of the dielectric constant, *ω* is the angular frequency. The Cole–Cole equation has been extensively used empirical equation so far. The abbreviations for the various samples shown in the figure legends are standard, and their full names and references were shown in previous reports [8,37,38]. Figure 2 shows the *τ*–*β* diagram for various aqueous systems that have been obtained so far, and in the present work, this entire diagram was used as a database in our analytical method.

The hyperbolic trajectory of the plots shown for each material is given by Equations (A1) and (A2). As the fractal dimension increases, the asymptote parallel to the ln *τ*-axis shifts upward in the *τ*–*β* diagram. Thus, trajectories for materials with higher HBN density and higher fractal dimension have been shown in the upper region of Figure 2 [7,8,22,37,38]. In contrast to conventional relaxation time distribution analysis using *β*-value, *τ*–*β* diagram analysis, which shows the relationship with average relaxation time, can obtain spatial structure information such as fractal dimension and spatial distribution of water structures. This interpretation is based on the experimental fact that the average relaxation time of 10 GHz processes decreases with increasing hydrogen-bond site density, providing a physical picture of relaxation times determined by hydrogen-bond rearrangement kinetics [20,21] and is supported by simulation results showing that HBN is broken and fragmented by solute molecules, particles, and their aggregates, leading to a reduction in the fractal dimension [8].

The HBN of each system exhibits different correlation lengths and fractal dimensions when aqueous solutions and dispersed systems are found at different locations on the *τ*–*β* diagram [7]. The fractal dimension of the dispersion system takes a small value corresponding to HBN fragmentation [7,8,22,37,38]. The curvature of the hyperbolic function corresponding to this low fractal dimension is large, and the measurable range of water content gives only partial hyperbolic trajectories, inevitably larger errors. Therefore, it is also reasonable that the experimental error is large for water structures with low fractal dimensions.

It has already been reported that the relationship between the HBN correlation length and the spatial scale of observation is also well understood for the hydrated collagen and gelatin trajectories in Figure 2 [37]. Although these two materials have the same primary structure, the secondary structure is different, and the structure of HBN is very different. HBN containing water molecules has a large correlation length and high fractal dimension because it penetrates between adjacent surfaces of the helical collagen molecule and its superhelix tropocollagen. On the other hand, HBNs fragmented by denatured coiled and aggregated gelatin molecules have shorter correlation lengths and lower fractal dimensions, as shown in the *τ*–*β* diagrams.

As mentioned earlier, dielectric measurements are often difficult to complete the entire trajectory on the *τ*–*β* diagram because of the characteristic shape of the hyperbolic function. Some more realistic cases of applied research using *τ*–*β* diagrams were then considered and recently reported [37,38,39,40]. Although the discussion is limited to fractal structures, more diverse and new applied research will be possible with the database of *τ*–*β* diagrams obtained so far, since Equations (A1) and (A2) simply require a general hopping model of charges. Following usages are possible in applications.

1. Single plot analysis for water structures for 10GHz process by comparison with database (Figure 2) [37,38].
2. Structured water analysis of relaxation processes other than the 10 GHz process, such as structured water observed in cement materials [33,35].
3. Ion migration in large-scale heterogeneous structures reflected by the Maxwell–Wagner effect of interfacial polarization [39].
4. Stability and variability of complex materials to assess states such as stability of states of matter and tissue homeostasis [40].

These recent applications of fractal analysis have become some of the most effective tools for a wide variety of wet materials, including biological tissues, allowing a simple and unambiguous understanding of water structure in complex materials. This study also uses analytical method #1 to investigate the structure and physical properties of several gels of different categories.

## 3. Results and Discussion

### 3.1. GHz Relaxation Process Observed for Cement Gels

**Figure 3:**
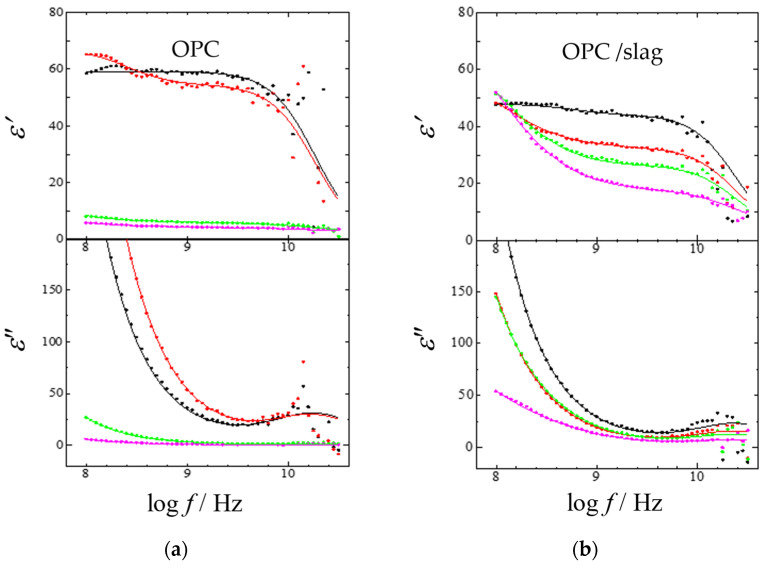
Changes in dielectric relaxation curves after preparations of (**a**) OPC and (**b**) OPC/slag pastes: immediately after preparation (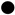), before (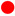) and after (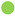) decreasing relaxation curve, and after one day (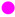). The solid lines were obtained from the fitting procedures.

Figure 3a shows the dielectric relaxation curves over time after preparing an ordinary Portland cement/water mixture (OPC paste) with a W/C ratio 0.60. Figure 3b also shows results for OPC/admixture paste, in which 30 wt% slag was used as an admixture of the powder. The relaxation curve showed a significant decrease about 10–20 h after sample preparation, while the sample exhibited a hardening called setting. Dielectric relaxation curves were obtained at four points: immediately after preparation, before and after decreasing, and one day later. Figure 3a clearly shows that the 10 GHz process decreases discontinuously in the decreasing process. This result implies that water molecules observed as 10 GHz processes exit the HBN and are included in the structured water in the cement gel [31,32,33,34,35,41,42,43]. Figure 3b shows that addition of slag reduces the discontinuous and abrupt changes in the relaxation curve. Several models of structural water have been proposed so far [44,45,46]. In the present study, however, we investigate water molecules that contribute to the relaxation process observed in the 10 GHz process, and the details of the structural water are not treated. The behavior of water is greatly affected by the addition of an admixture [47,48,49].

To examine the observed relaxation curves in more detail, the relaxation parameters for each relaxation process involved were obtained from the relaxation curve fitting procedures by the following Equation:

(2) $$\varepsilon^{*}=\varepsilon_{\infty }+\overset{h,\;m,\;l}{\underset{k}{\sum }}\frac{\Delta \varepsilon_{k}}{1+{\left(j\omega \tau_{k}\right)}^{\beta_{k}}}+\frac{\sigma }{j\omega \varepsilon_{0}}\;,$$

where *σ* is the direct current (dc) conductivity, and ε_0_ is the vacuum permittivity. Equation (2) contains three Cole–Cole relaxation functions [36] for *h*-, *m*-, and *l*-process denoted from the higher frequency side and a dc conductivity term. The solid lines shown in Figure 3 were drawn as the calculated dielectric relaxation curve obtained from the fitting procedure. The experimental data were obtained with good precision up to frequencies near 10 GHz.

**Figure 4:**
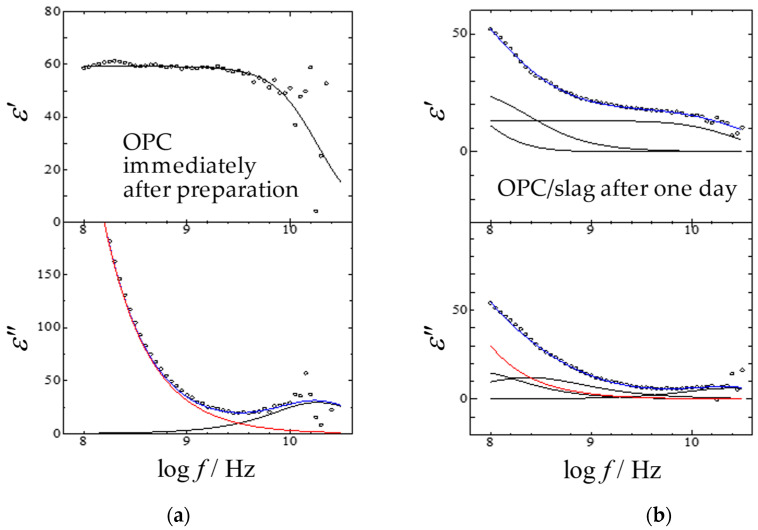
The curve-fitting results and frequency dependence of complex permittivity for aqueous mixtures with the W/C ratio of 60% for (**a**) OPC paste obtained immediately after the preparation and for (**b**) OPC/slag paste as 30 wt% slags obtained after one day. Blue lines are the sum of each process, and red lines are dc contributions. The solid lines were obtained from the fitting procedures.

Figure 4 shows the relaxation curves of the OPC paste immediately after preparation and the OPC/slag paste after one day, as examples of the fitting procedure by Equation (2), and the contributions separated for each process. The *m*- and *l*-processes were not yet shown in Figure 4a, but they increased as the *h*-process (10 GHz process) decreased and are shown in Figure 4b.

**Figure 5:**
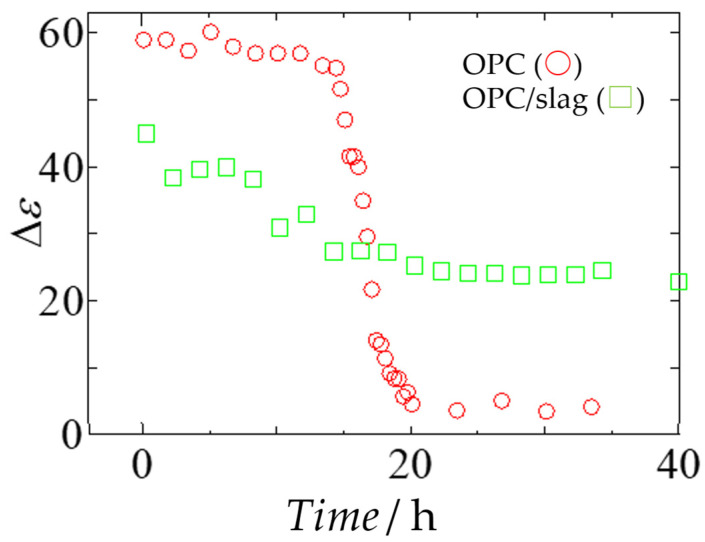
Time dependence of the relaxation strength after OPC (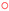) and OPC/slag (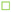) paste preparation, respectively.

Figure 5 shows the relaxation strength of OPC paste and OPC/slag paste over time, clearly implying the reduction of the *h*-process due to cement hydration [31]. The smaller relaxation strength obtained immediately after preparation of the OPC/slag paste is because the admixture lowers the number density of water molecules. The OPC paste did not change significantly until 15 h after sample preparation but decreased during 5 h from 15 h to 20 h after the preparation. Although the addition of slag continued to reduce the relaxation strength for a long time, up to 30 h after preparation, it was not as low as that shown for the OPC paste.

These results indicate that cement and admixture particles retain moisture even after sample preparation. Slow dynamics of water due to hydration of the admixture powder reduces the water content observed as the *h*-process from the beginning, whereas reduction due to cement gel formation occurs during 15–20 h. Therefore, Figure 5 suggests that the water retained around the admixture was gradually supplied to the OPC powders and then hydrated.

**Figure 6:**
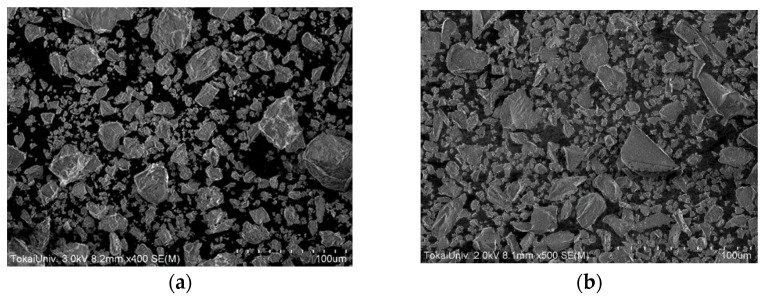
Electron micrographs of (**a**) OPC and (**b**) slag powders.

**Figure 7:**
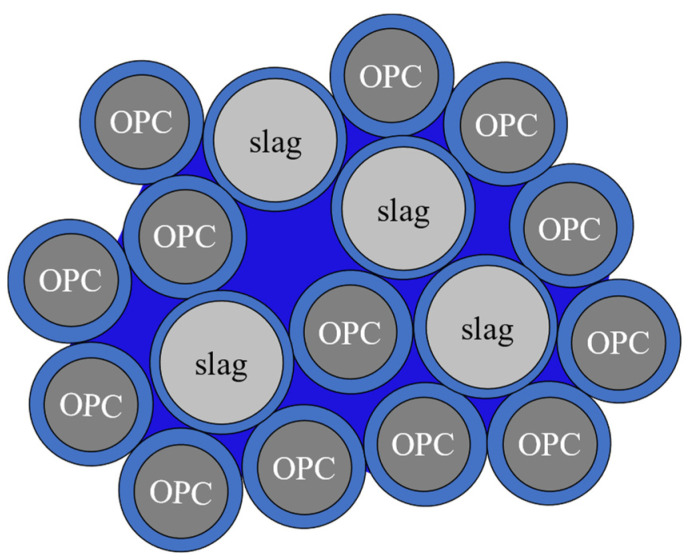
A hydration model of cement and slag particles with structured water on particle surfaces (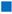) and distributable interparticle storage water (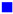).

Figure 6 shows electron micrographs of the OPC and slag particles used in the present work. It was shown that both particle sizes are widely distributed. Figure 7 shows a simplified water retention model by OPC and slag particles. The amount of water retained among particles depends on the particle size, and the superposition of the effects of both particle size and particle hydration properties determines the time dependence shown in Figure 5. A more detailed model of these molecular mechanisms from the time dependence will be reported separately in the future, along with systematic measurements of OPC/admixture pastes.

### 3.2. Diagrams of the Relaxation Time vs. Its Distribution Parameter

#### 3.2.1. Polymer Gels

We have already reported the results of dielectric spectroscopy and nuclear magnetic resonance measurements of polyacrylamide (PAAm) gels using aqueous solutions of acetone, 1.4-dioxane, and dimethylsulfoxide (DMSO), respectively [23,24,27]. Results of several globular protein aqueous solutions have also been reported, with results indicating gelation at higher concentrations [22]. Figure 2 shows the trajectory of a PAAm gel plot showing the volume phase transition phenomenon [1,2,50,51,52,53,54] in the upper area of the *τ*–*β* diagram as a water structure with high fractal dimensions like various synthetic polymer aqueous solutions. On the other hand, the trajectory of the plot obtained for the globular protein aqueous solution is shown in the lower left area as the structure of water with a low fractal dimension.

In the study of the volume phase transition of PAAm gels [50,51,52,53,54], the relaxation time of the h-process obtained by dielectric measurements and the diffusion coefficient of water molecules obtained by NMR diffusion measurements were expressed by the scaling concept and the universal dynamic behavior was analyzed [2,9,10,11,12,13]. The relaxation time behavior resembled the concentration dependence of normal aqueous systems, but the fully shrunk gel exhibited anomalous behavior with a smaller-than-expected increase in relaxation time. This result was interpreted as the mixed solvent composition deviating from the macroscopic value and higher water content [23,24,27].

**Figure 8:**
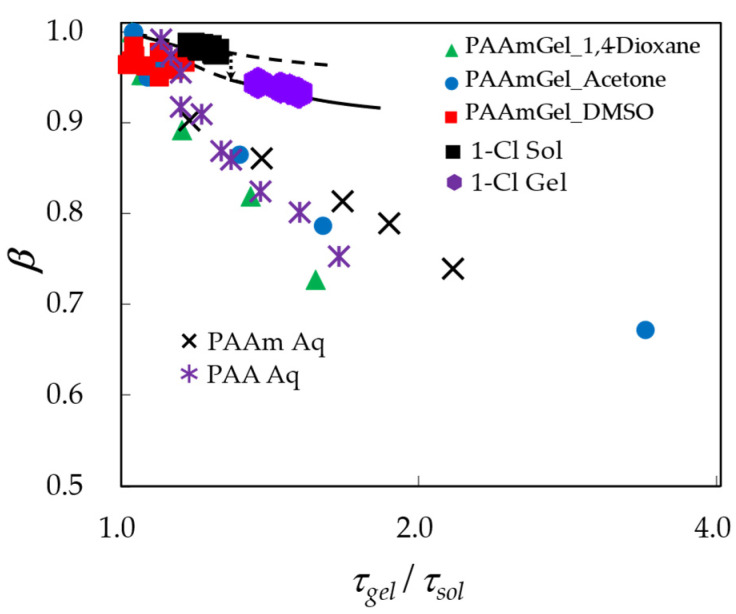
The *τ*–*β* diagram for PAAm gels with aqueous mixtures of 1.4-dioxane (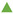), acetone (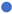), and DMSO (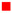). The results obtained for 1-Cl in sol (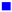) and gel (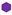) states are also shown. (Adapted from Saito et al. [27] and Kundu et al. [29]).

Since it is difficult to distinguish between the scaling behavior of solvent molecules confined in the gel network and the normal concentration-dependent slow dynamic behavior of solvent molecules, except in regions of strong contraction, we used the *τ*–*β* diagram shown in Figure 8 reproduced from our previous report [37,38] for the fractal analysis. For comparison, the results of PAAm and an aqueous solution of PAA considering the possibility of hydrolysis are also shown in Figure 8. Though the volume range is dependent on the solvent, in the case of the gel, the trajectory of the plot shifts toward the lower left in the figure compared to the aqueous system, indicating that the HBN is broken and has a low fractal dimension water structure. The plot of the most contracted gel obtained in an aqueous acetone solution cannot be realized with the aqueous system due to the precipitation. The position of the plot is shifted in the water-rich direction on the right side of the screen.

On the other hand, these intermediate region plots show that the curves in the figures do not differ significantly, indicating that the curves are not fundamentally different in shape. This fact implies that the hydrogen-bonding network of the solvent is not fragmented by gelation. This is probably due to the fact that the polymer chain network and the hydrogen-bonded network form an interpenetrating network, and the water in collagen does not degrade the hydrogen-bonded network more than the water in gelatin [37,38]. Therefore, the characteristics of the sol state system remain even after gelation, and the increase in heterogeneity due to the formation of cross-linking points becomes the characteristic of the gel structure.

Depending on the structure of the cross-linking points, the water structure can be more heterogeneous, and the trajectory of the plot on the *τ*–*β* diagram shows a larger discontinuity at the gelation point. However, for globular protein gels, HBN is already broken by a single protein molecule before gelation and is only part of the hyperbolic trajectory along the *β*-axis on the far left of the *τ*–*β* diagram. Therefore, discontinuities at the gelation point of globular proteins cannot be clearly seen in the *τ*–*β* diagram [22].

#### 3.2.2. Supramolecular Gels

Figure 8 also shows the sol-gel transition results for an aqueous solution of poly [pyridinium-1,4-diiriminocarbonyl-1,4-phenylene-methylene chloride] (1-Cl) used as an oligomeric electrolyte gelling agent. The concentration dependence of the relaxation parameter analyzed for the 10 GHz process obtained from dielectric spectroscopy measurements of 2–20 g/L 1-Cl/water mixtures at 25 °C is shown. The measurement error is small compared to measurements of polymer gels with large volume changes, and a clear gelation-associated discontinuity is observed at the critical concentration of 6.3 g/L [28,29]. Electrolytic oligomers crosslinked via ions and water molecules exhibit characteristic properties of both polymers and supramolecular gels. The relaxation strength of the *h*-process also causes a decrease at the critical concentration because the water molecules involved in cross-linking have limited mobility [29].

A characteristic feature of the 1-Cl gelator as a polymer gel is that the trajectory on the *τ*–*β* diagram appears in the region of the synthetic polymer gels in Figure 8. It is also interesting to notice that the trajectory on the *τ*–*β* diagram clearly shifts to the lower left due to the sol-gel transition. This property means that the water molecules contained in the cross-link of oligomers form larger cross-linked structures and break the HBN of free water, contributing to the 10 GHz process.

Recently, we reported how supramolecular gelators aggregate to form rod micelles, elongate into fibres, and finally, cross-link to form a gel, while a 5 wt% N-oleyl lactobionamide/water system is cooled from high temperature [30]. We performed dielectric spectroscopy measurements to investigate the molecular mechanism. At 53 °C, which is higher than the macroscopic gelation temperature of 37.8 °C obtained by the falling ball method, a characteristic change in water structures due to aggregation of the gelator was observed [55,56,57,58,59,60,61]. Although the relaxation time distribution becomes entirely narrower due to the structure formation during the cooling process, the distribution becomes relatively large when the rod micelles elongate and also the cross-linking starts.

**Figure 9:**
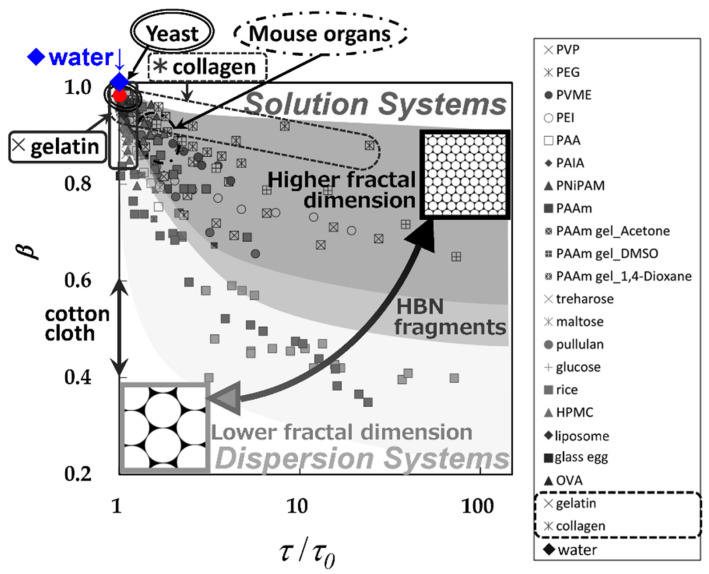
Plots for supramolecular gel obtained from an aqueous solution of 5 wt% N-oleyl lactobionamide/water mixture (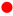) [30] and pure water (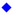) are shown in our database of *τ*–*β* diagrams. (Adapted from Yagihara et al. [38]).

The increase in relaxation time distribution indicates fluctuations in the spatial distribution of water molecules due to HBN breaking with a decrease in fractal dimension, but not much change compared to our database on the *τ*–*β* diagram in Figure 9. In particular, the change in the aggregation structure of water molecules seems to be quite smaller than that expected due to the temperature change larger than 45°. This change, which is also smaller despite the macroscopic sol-gel transition of the whole system, indicates that the water structure around the low-molecular-weight gelator did not change significantly at the observed scale. This is because the gelators have an aggregation structure even in the high-temperature region in which gelators were considered to be initialized, and the water structure is disturbed over a long period of time due to the formation of a larger-scale structure during the subsequent cooling process. This result indicates that the local water structure reflected in the 10 GHz process is not disturbed much by the large-scale structuring.

Regarding the molecular mobility and fluctuations, even in the same category of dispersed systems, the water structure fluctuations around the liposomes observed during the heating process of 3 wt% liposome suspensions [62,63] are significantly higher than those for the supramolecular gels. The rod micelle formation might be interpreted as stabilizing the surrounding water structures contributing to the 10 GHz processes observed on short timescales and making the spatial distribution of HBN more uniform. The more stable spatial distribution of water structures is already realized during rod micelles structure formation as a precursor to the gelation in the sol state.

#### 3.2.3. Cement Gels

**Figure 10:**
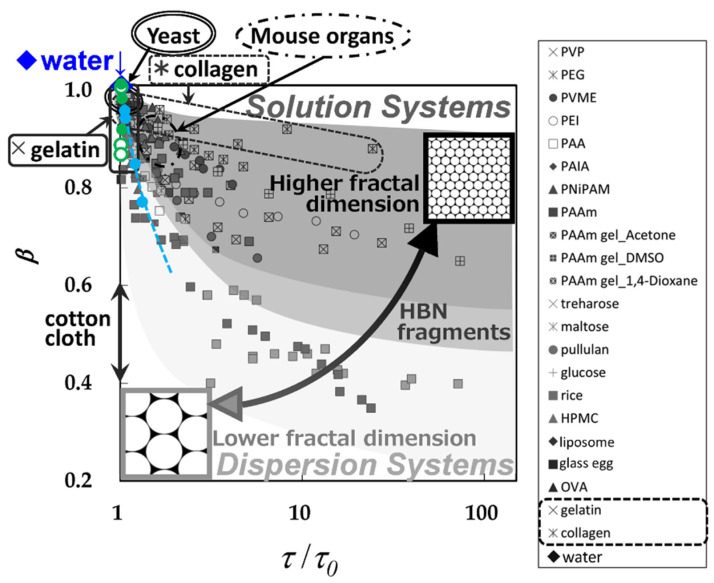
Plots obtained for OPC paste (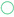) and OPC/slag paste (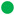) with a W/C ratio of 0.60 are shown with our database of *τ*–*β* diagrams. The dashed line is obtained for the W/C ratio dependence of OPC paste (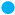) [34]. (Adapted from Yagihara et al. [38]).

Figure 10 shows two series of data of *τ*–*β* diagram obtained for cement gels with our database. The plots indicated by the dashed line were obtained as the W/C ratio dependence of the relaxation parameters of the 10 GHz process for OPC paste 5 h after the preparation and before decreasing relaxation curves. The trajectory shows plots shifted to the lower right with decreasing W/C ratio, a characteristic behavior of typical dispersion systems. This means that cement particles and their hydration breaks the HBN, and the fragmentation of HBN reduces the fractal dimension of the cement gel.

Figure 10 also shows plots obtained for the OPC and OPC/slag pastes when prepared with a W/C ratio of 60%, just before and after decreasing relaxation curves and one day after preparation. The plot shifts to the lower right with time, showing the same trend as the decrease in the W/C ratio. This result indicates that the free water in the cement paste changed to structural water observed in the low-frequency region, and the free water component decreased [31,32,33,34,35]. For HBN dynamics involving free water, the water molecules act as plasticizers. This plasticizer effect can also be interpreted by the hydrogen bond density dependence of the relaxation time.

Plots for OPC/slag paste were shown in the upper area on the *τ*–*β* diagram compared to those for OPC paste. This result indicates the existence of more free water there since the decrease in the relaxation curves gradually occurs with admixture particles for a longer time. Water held among admixture particles is gradually supplied to hydrate OPC particles, as shown in Figure 5.

These cement gels are representative dispersions of fragmented HBN, and a distinguishing property of gelation is the reduction in the number density of water molecules reflected in the 10 GHz process.

## 4. Conclusions

Using *τ*–*β* diagram obtained by dielectric spectroscopy, various gelation mechanisms were investigated from the viewpoint of water structures. Details of the molecular mechanism for each gelation, such as the interpenetrating network of polymer chains and HBN of water molecules in polymer gels, the formation of precursor structures for gelation of supramolecular gels, and the relationship between cement setting and the plasticizer effect by free water were interpreted from water structures. The common feature of these molecular mechanisms is not the specific region on the *τ*–*β* diagram, but the change associated with gelation within the region characteristic of each gel. The degeneracy of the fractal dimension of the spatial distribution of water was identified as a common feature of various gelation mechanisms. The collective structure and state of water molecules commonly involved in each system implied that the HBN formed by water and host molecules was further broken during the gelation process, resulting in a fragmented spatial distribution. It is essentially impossible to determine the spatial distribution of water molecules from relaxation data that represent only time correlations of the structure and physical properties. However, using the universal analysis presented in this work, we find that we can obtain information about the fractal structure of the spatial distribution of water molecules, even in any kind of gel.

It is expected that this new universal analysis of water structures will enable us to characterize the water structure and evaluate the gelation process in various kinds of aqueous gel systems. By clarifying the relationship between physical properties such as strength and stability of various gels and the water structures, the application of universal analysis based on the water structures will expand to a wider range of research subjects and technical fields. The analytical method for various gel structures and gelation mechanisms from the water structures presented in this study are easily applied to developments of evaluation systems for food quality [7,22,64,65], pathology and health of biological tissues [7,8,38,66], concrete constructions [31,32,35,67,68], and so on. It is expected that these new evaluation systems will be put into practical use. Furthermore, if suitable BDS systems capable of performing dielectric spectroscopy measurements for any moist materials and a sufficient database are available, a greater variety of substances and organisms treated in diverse fields can be analyzed. Practical application of a new evaluation system suitable for these uses is also expected.

## 5. Materials and Methods

### 5.1. Sample Preparations

Regarding polymer gels and supramolecular gels, previously reported data are newly analyzed. PAAm gels were prepared using aqueous solutions of acetone, 1,4-dioxane, and DMSO as solvents and varying the composition ratio by adding each organic solvent to water. Pure water was produced with a water purifier (Direct-Q^®^ UV3 Water Purification System, Merck Millipore Corporation, Burlington, MA, USA) and had a resistivity of 18.3 MΩ cm. We determined the relaxation parameters for the 10 GHz processes obtained both inside and outside the gel solvent mixtures. Details of sample pretreatment and dielectric measurements are described in our previous paper [27].

As an example of a supramolecular gel, the fractal water structure analysis is applied to the results of dielectric spectroscopy measurements of gelation during the cooling process of a 5 wt% N-oleyllactobionamide/water system initialized at high temperatures. The details of the experiments and results are described in another report [30].

Powder samples of standard OPC and slag were kindly provided by Taiheiyo Consultant Co., Ltd. (Sakura, Chiba, Japan). A previous PMEA analysis of mortar prepared with a standard sample of the same kind as this OPC sample showed that the chemical composition obtained with oxygen indicated a usual composition of a normal OPC sample [35]. Ultra-pure water products (Millipore, MILLI-Q Lab, Darmstadt, Germany) were used to provide distilled and deionized water with an electric conductivity lower than 18.3 S. Milli-Q water was used to make cement-paste samples with a range of W/C ratios between 0.40 and 0.70.

As another sample of admixture-introduced cement paste, a powder mixture of OPC and slag with an S/C ratio of 0.5 and Milli-Q water with a W/C ratio of 0.6 was mixed.

### 5.2. Dielectric Measurements

Complex permittivity of the cement-paste samples with W/C ratios of 0.40, 0.50, 0.60, and 0.70 was measured by the time domain reflectometry (TDR) method [62,63,66,69,70,71,72,73] in the frequency range between 750 MHz and 20 GHz. During the isothermal cure at 25.0 °C, the temperature was managed via a temperature-controlled bath with a digital multimeter.

The pulse generator in the sampling head (HP54121A, Hewlett-Packard, Colorado Springs, CO, USA) generated a step pulse with a rise time of 35 ps and a pulse height of 200 mV. A step pulse was incident on the sample through a coaxial cable, and the returning reflected pulse was detected. The reflected wave was digitized with a digital oscilloscope mainframe (HP54121B, Hewlett-Packard, Colorado Springs, CO, USA), and the polarization information of the constituent molecules was analyzed from the averaged pulse signal. Complex permittivity values are obtained from analyses involving Fourier transforms.

Coaxial electrodes are often used for TDR measurements of gel-like materials. The flat end of the electrode is brought into contact with the sample surface, and the leakage field from the open end is used for measurement. The appropriate shape and diameter of the electrodes are designed so that the penetration depth of the electric field used for the measurement is optimal for the purpose [7,8,22,24,26,27,66]. Details of the dielectric relaxation measurement procedure, especially for cement materials, have been reported in previous papers [31,32,33,34,35].

The TDR method was also used to measure the amount of cement admixture added, the temperature was adjusted to 25 °C ± 0.3, and the measurement frequency was averaged 512 times from 100 ps to over 1 ns. Air, Milli-Q water, and acetone were used as reference substances. The cement paste was prepared in a 5 mL tube and the tube was plugged with a rubber stopper during the measurement to minimize evaporation of water during the measurement.

## Data Availability

Not applicable.

## Appendix A. *τ*–*β* Diagram

Ryabov et al. described the relationship between the Cole–Cole parameters, viz., relaxation time distribution *β* and the relaxation time *τ* defined by Equation (1) in the fractal structure expression [14,15,16]:

(A1) $$\;\beta =\frac{D}{2}\frac{\ln\left(\tau \omega_{S}\right)}{\ln\left(\tau /\tau_{0}\right)},$$

where, *D* represents the fractal dimension of the point set where relaxing units are interacting with the statistical reservoir, and *τ*_0_ represents the cutoff time of the scaling in the time domain.

(A2) $$\;\omega_{S}=2d_{E}G^{2/D}D_{S}/R_{0}^{2}$$

is the self-diffusion process’ characteristic frequency where *d_E_* represents the Euclidean dimension, *D_S_* represents the self-diffusion coefficient, *R*_0_ is the cutoff size of the scaling in the space, and *G* is a geometrical coefficient close to unity.

Equations (A1) and (A2) have been explored in detail in a series of papers by Feldman et al. [14,15,16,17,18,19], where *β* is reported to be a hyperbolic general function of log *τ* [17]. The proper values and physical meanings of all the constants appearing in these equations are also fully explained in this series. The validity of Figure 2, which deals with the analytical method used in the present paper, is ensured by Equations (A1) and (A2).

The value of the fractal dimension is 1–1.5 for polymer solutions, but it is smaller than 1 for dispersion systems such as protein solutions and suspensions, and the error is large [7]. Therefore, it is appropriate to discuss the value of the fractal dimension not as a precise value or an absolute value but as a relative value or variation [8].

The *τ−β* diagram makes it evident that plots and trajectories derived from the dependence of the water content are localized in each region for solution or dispersion systems. At the outset of the analytical procedure, it was noted that quite small values around 0.1 and the significant errors around 100% of the fractal dimension obtained for dispersion systems like protein/water mixtures were issues. A fractal dimension adjustment parameter was set to 2 in real applications of Equation (A1), following a two-dimensional hydrogen bond network. The fractal dimension at 0.1 remains modest even if the correction parameter is fixed again.
